# Unique Presentation and Perioperative Management of a Giant Pheochromocytoma

**DOI:** 10.1210/jcemcr/luad065

**Published:** 2023-06-09

**Authors:** Nicolas Villarraga, Gurunanthan Palani, Michael Risk, Shalamar Sibley

**Affiliations:** University of Minnesota School of Medicine, Minneapolis, MN 55415, USA; Department of Medicine, Division of Endocrinology, University of Minnesota, Minneapolis, MN 55415, USA; Department of Surgery, Division of Urology, Minneapolis VA Health Care System, Minneapolis, MN 55417, USA; Department of Medicine, Division of Endocrinology, Minneapolis VA Health Care System, Minneapolis, MN 55417, USA; Department of Medicine, Division of Endocrinology, University of Minnesota, Minneapolis, MN 55415, USA

**Keywords:** giant pheochromocytoma, nephrectomy, adrenalectomy, management

## Abstract

Pheochromocytomas are neuroendocrine tumors that arise from chromaffin cells in the adrenal medulla. Giant pheochromocytomas commonly measure greater than 7 to 10 cm, and their incidence and presentation is not well known. We present a case of a 62-year-old female with a giant 15.9 cm cystic pheochromocytoma. The patient was medically managed with oral phenoxybenzamine solution dose 4 times greater than average and was treated with a radical left nephrectomy and adrenalectomy. This case offers insight into the clinical presentation of giant pheochromocytomas and the unique challenges they present both medically and surgically.

## Introduction

The overall annual incidence of pheochromocytomas, neuroendocrine tumors that arise from chromaffin cells in the adrenal medulla, is 2 to 8 per million persons per year [1]. Classic symptoms include paroxysmal headaches, diaphoresis, and tachycardia, with approximately one-half of patients experiencing paroxysmal hypertension [1]. Incidental discovery of pheochromocytomas is becoming more common with widespread imaging [1]. Typical preoperative management consists of adequate initial α-blockade followed by β-adrenergic blockade to help prevent tachycardia. Typically, giant pheochromocytomas measure greater than 7 to 10 cm [1, 2]. Currently, incidence and presentation of giant pheochromocytomas is not well known, with only a few cases discussed in case reports. Further, preoperative and surgical treatment of giant pheochromocytomas is not well established. We present a case of a 62-year-old female with a giant 15.9 cm cystic pheochromocytoma that had complicated preoperative management and was treated with radical left nephrectomy and adrenalectomy.

## Case Presentation

The endocrine service was consulted for preoperative management of a pheochromocytoma in a 62-year-old female. Her past medical history included uncontrolled stage 2 hypertension, noncompliant with the home medication (lisinopril), and uncontrolled type 1 diabetes. The patient had no family history of pheochromocytoma or other endocrine tumor. Over the 18 months prior to admission, the patient had experienced an 80-lb weight loss along with nausea, vomiting, episodes of paroxysmal headaches, tachycardia, and 1 episode of hypertensive emergency.

## Diagnostic Assessment

**Figure 1.**
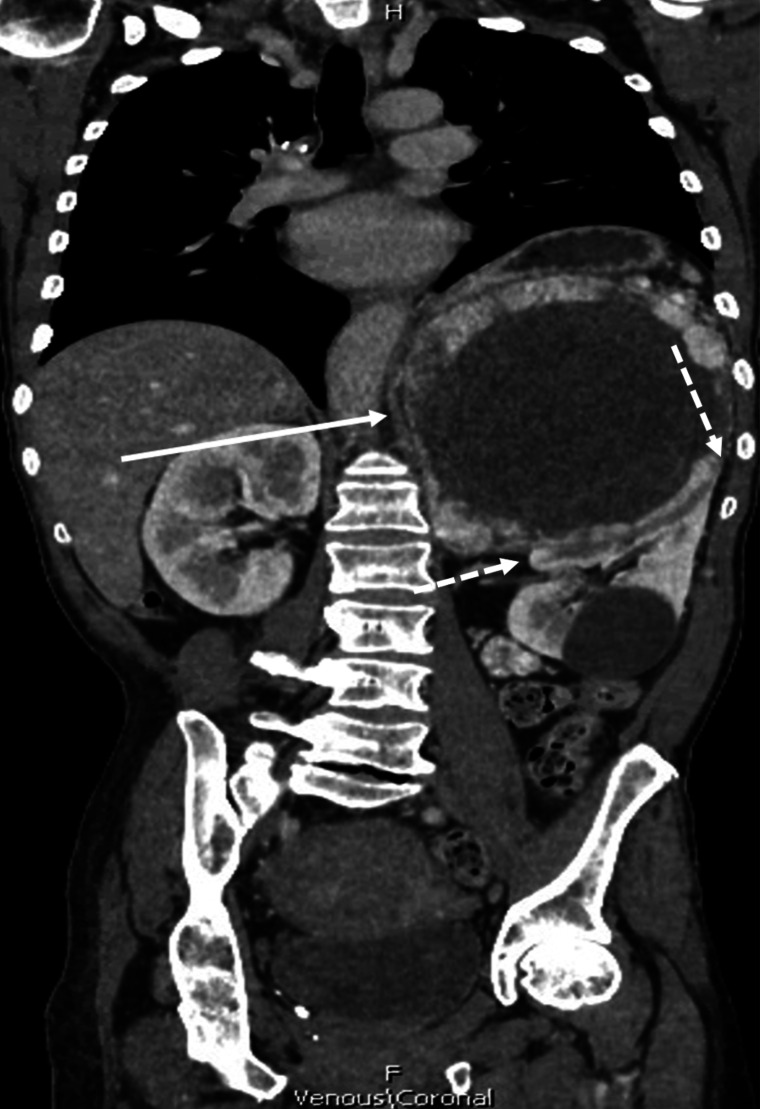
Abdominal computed tomography scan (coronal image) showed a centrally necrotic giant pheochromocytoma (solid arrow) overlying the left kidney, measuring 15.9 × 12.7 × 14.7 cm. “Claw sign” can be seen as the tissue circumscribing the bottom of the giant pheochromocytomas, indicated by dashed arrows.

**Figure 2.**
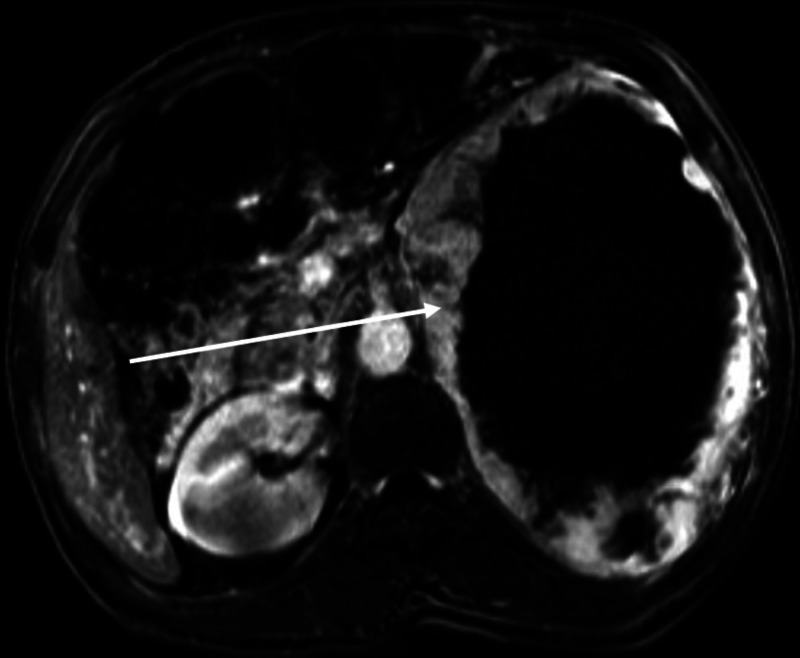
Abdominal magnetic resonance imaging (axial image) showed a large centrally necrotic, heterogeneous, and peripherally enhancing mass (arrow) in the left upper abdomen that measured 15.9 × 12.7 × 14.7 cm.

On admission, the patient was hypertensive and tachycardic. She denied any headaches, diaphoresis, or palpitations. Computed tomography (CT) scan revealed a centrally necrotic mass overlying the left kidney, measuring 15.9 × 12.7 × 14.7 cm (Fig. 1). A magnetic resonance imaging (MRI) scan was obtained and revealed a large centrally necrotic, heterogeneous, peripherally enhancing mass with claw sign at the superior pole of the left kidney suggesting renal origin. The mass was relatively well circumscribed and hypointense on T2 (Fig. 2). The admitting team had an initial diagnosis of a tumor of renal origin, given the appearance of claw sign on the left kidney. However, there was still concern for a tumor arising from the adrenal gland and so they screened for pheochromocytoma. Laboratory tests revealed total plasma metanephrines >40 000 pg/mL (>210 400 pmol/L) (reference range ≤205 pg/mL), plasma normetanephrines >20 000 pg/mL (>109 200 pmol/L) (reference range ≤148), and plasma metanephrines 57 pg/mL (289 pmol/L) (reference range ≤57). No preoperative nuclear images to evaluate for metastasis were performed.

## Treatment

The patient was started on phenoxybenzamine with a goal for sitting blood pressure < 120/79 mmHg. She increased sodium intake with sodium chloride tablets and normal saline flushes through her tube feed to reach a total goal of 5 grams daily. Over the following days, the blood pressure medication regimen was titrated aggressively to control blood pressure prior to surgery (Table 1).

**Table 1.** Summary of medication course

Day	Phenoxybenzamine	Amlodipine	Metoprolol	Metoprolol succinate
1	10 mg/BID; oral	5 mg; oral	—	—
2	20 mg/BID; oral	5 mg; oral	—	—
3	20 mg/BID; oral	—	25 mg/BID; oral	—
4	20 mg/TID; oral	—	25 mg/BID; oral	—
7	30 mg/TID; oral	—	25 mg/BID; oral	—
9	30 mg/TID; oral	—	50 mg/BID; oral	—
12	40 mg/TID; oral	—	—	100 mg; oral
13	140 mg; oral	—	—	100 mg; oral
14	160 mg; NJ tube solution*^a^*	—	—	150 mg; oral
15	180 mg; NJ tube solution	5 mg; oral	—	150 mg; oral
17	240 mg; NJ tube solution	5 mg; oral	—	200 mg; oral

Abbreviations: BID, twice daily; NG, nasogastric; NJ, nasojejunal; TID, 3 times daily.

a Solution made out of phenoxybenzamine capsules, propylene glycol 1%, citric acid 0.15%, and distilled water.

She underwent tumor resection. Preoperative α-adrenergic blockade followed by β-adrenergic blockade successfully managed blood pressure throughout surgery. The patient's prior code status of DNI/DNR was suspended for the surgical intervention and continued through the 4 hours immediately after the operation. During surgery, the patient's systolic blood pressures ranged between 90 seconds and 130 seconds, and diastolic blood pressures ranged between 60 seconds and 80 seconds, with pulse ranging between 70 and 90 seconds. In surgery, the pheochromocytoma was found to be adhered to the kidney and a plane could not be developed to allow for separation. Due to concern for puncturing the tumor intraoperatively, surgeons performed a radical left nephrectomy and adrenalectomy. The anesthesia report included fluid support along with 8 units packed red blood cells, given for intraoperative blood loss combined with her history of chronic iron deficiency anemia. In the immediate postoperative period, the patient's phenoxybenzamine, metoprolol, and amlodipine were discontinued, and she was monitored closely for labile blood pressures and glycemic control. Nadir glucose postoperatively was 66 mg/dL (3.66 mmol/L); hemoglobin dropped to 6.5 g/dL (65 g/L); and blood pressures were initially labile with pressor, transfusion, and fluid support required over about 24 to 36 hours.

## Outcome and Follow-up

The tumor was taken to pathology for analysis. Per the pathology report, the pericolonic mass was described as well circumscribed with negative margins. On the microscopic exam, sections were composed of solid, trabecular, and focally nested arrangements of neoplastic cells, pleomorphic with granular cytoplasm, and focal nucleoli. The majority of the tumor was noted to be cystic, and the mass had a well-defined capsule. Immunohistochemical cellular staining results were as follows: chromogranin (+), synaptophysin (+), CD10(+), S100(+), AE1/AE3 (−), CK7(−), PAX8(−), vimentin (−). The grading system for adrenal pheochromocytoma and paraganglioma and pheochromocytoma of the adrenal gland scaled score grading systems were not performed as these systems are not routinely applied by our pathologists.

Unfortunately, the patient did not keep her endocrine follow-up appointment after discharge and therefore did not undergo subsequent tumor staging and genetic testing. Since then, she has followed up somewhat with primary care but not endocrine. Plasma metanephrines about 6 weeks postoperatively were normal. Abdominal/pelvic CT scan was performed to evaluate drain placement and resolution of a fluid collection in the nephrectomy bed, which showed resolution of the fluid and no evidence of recurrence or metastasis within the abdomen or pelvis. Her primary care has recently obtained plasma metanephrines, and they were within normal ranges. She is now establishing care with our endocrine department and will continue biochemical screening and additional longer-term follow-up. She had declined genetic testing, but this will be readdressed. She currently requires only 1 medication for control of hypertension (nifedipine).

## Discussion

We report a case of a giant pheochromocytoma with a diameter size of 15.9 cm. There is no clear cutoff of giant pheochromocytoma. However, current literature suggests that pheochromocytomas greater than 7 to 10 cm are considered giant [1, 2]. Given their rarity, the spectrum of presentation and clinical findings of these large tumors are not well characterized. An extensive review by Maharaj et al analyzed the 36 giant pheochromocytomas greater than 10 cm and found that only 6 cases presented with classic symptoms and 12 cases were asymptomatic. Our patient had a known history of classic pheochromocytoma symptoms, placing her in the minority of presentations reported for patients with giant pheochromocytomas. Biochemical findings for giant pheochromocytomas may also differ greatly among cases. A retrospective study conducted by Gruber et al showed that pheochromocytomas >4 cm had total plasma metanephrines 15.4 times greater than the upper limit of normal, whereas total plasma metanephrines in tumors <4 cm were only 3.4 times greater [3]. Interestingly, the third largest pheochromocytoma (the largest did not have catecholamines assessed), reported by Costa et al and measuring 30 cm revealed normal catecholamines [4]. The second largest pheochromocytoma to date, reported by Arikan et al, demonstrated a giant cystic pheochromocytoma (size of 30 × 23 cm) with metanephrine level of 371 pg/mL (1951pmol/L) and normetanephrine of 7795 pg/mL (42 561 pmol/L), in comparison to our patient who had total metanephrines >40 000 pg/mL (>210 400 pmol/L) normetanephrines >20 000 pg/mL (>109 200 pmol/L), and metanephrines 57 pg/mL (289 pmol/L) [5]. Taken together, these cases illustrate how giant pheochromocytomas may differ considerably in their presentation and clinical findings compared to pheochromocytomas <7 to 10 cm. More information on the spectrum of findings, management, and clinical outcomes for giant pheochromocytomas is needed. Our case adds to the clinical presentation of giant pheochromocytomas.

Preoperative preparation was challenging in this case. Following the Endocrine Society guidelines, phenoxybenzamine can be started on 10 mg twice daily and can be titrated if/as needed to reach a final dose of around 1 mg/kg/d [6], with doses typically reaching an average of 44 mg/day [7]. Our patient was started on 10 mg twice daily and aggressively titrated over the course of 3 weeks to 240 mg/day in order to reach preoperative blood pressure goals. At the time of admission, our patient weighed 50 kg. Thus, she received over 4 times the typical per kg dose. Gruber et al reported that pheochromocytomas with size of >4 cm need a greater cumulative dose of phenoxybenzamine compared to pheochromocytoma <4 cm (420 mg vs 335 mg, *P*-value .02). Further, our patient was also treated with a calcium channel blocker and a beta-adrenergic blocker, after advancing alpha-blockade, as recommended by the Endocrine Society [6]. An additional complicating factor was our patient's inability to consistently take and absorb oral medications; nausea and vomiting prompted insertion of a nasogastric (NG) and, subsequently, nasojejunal (NJ) tube. Due to concerns about inadequate absorption of phenoxybenzamine hydrochloride delivery through NG tube resulting from adherence to the NG tubing, the pharmacy prepared a 60 mg/30 mL (totaling 240 mg/day) suspension made of phenoxybenzamine capsules, propylene glycol 1%, citric acid 0.15%, and distilled water for delivery via NJ tube. There has been only 1 reported case from Kinney and colleagues that used 240 mg of phenoxybenzamine daily for blood pressure, and it is unknown how many cases have used an oral solution. Furthermore, Kinney and colleagues did not report the use of calcium channel blockers in conjunction with phenoxybenzamine, suggesting that our patient required more aggressive management compared to that case. Our case provides further experience regarding successful preoperative management of giant pheochromocytomas and can aid other physicians who encounter this challenging condition.

CT and MRI are common imaging modalities used to locate pheochromocytomas after medical diagnosis. Our patient received an MRI that showed a T2, hypointense tumor with apparent claw sign at the superior pole of the left kidney. These 2 findings go against typical pheochromocytoma findings on imaging. Pheochromocytomas are typically hyperintense on T2 [8]. Furthermore, MRI imaging noted the claw sign on the left kidney. The “claw sign” usually indicates that normal parenchymal tissue of the organ of origin is wrapping around a developing tumor [9]. Thus, it was initially believed this patient's tumor was of renal origin. To date, there are few reported cases in which a pheochromocytoma have been misdiagnosed as renal mass [9]. Thus, our case provides support for masses that appear to be in the upper pole of the kidney to be screened for a pheochromocytoma.

Definitive treatment of pheochromocytomas includes adrenalectomy. During surgery, it was found that the tumor was significantly adhered to the kidney, and the surgeons could not locate a safe dissection plane between the tumor and kidney without the risk of rupturing the tumor. Thus, an adrenalectomy and nephrectomy were performed. Literature on adrenalectomy with nephrectomy for pheochromocytoma is limited, and nephrectomy has been mainly done in cases of renal invasion or involvement of renal artery or aorta [10].

There are important postoperative considerations for patients with confirmed pheochromocytomas. Unfortunately, our patient was lost to follow-up and did not receive genetic testing or whole-body imaging. She is establishing outpatient endocrine care, and we will readdress screening and genetic testing. Genetic testing is recommended for all patients with pheochromocytomas, even those with apparently sporadic tumors like our patient [6]. Staging should occur to evaluate for metastatic disease; CT, MRI, and nuclear imaging have all been employed [6].

In conclusion, our case is unique among other reported giant pheochromocytoma cases due to its diagnostic imaging challenges, complex preoperative treatment challenges, which included high dose alpha-blockade and novel administration of phenoxybenzamine, and completion of a radical left nephrectomy and adrenalectomy. This case offers novel information for future physicians who encounter these rare tumors.

## Learning Points

- Giant pheochromocytomas are rare, and their presentation is different than average-sized pheochromocytomas.
- Medical management of giant pheochromocytomas may need aggressive treatement with phenoxybenzamine doses reaching 240 mg per day.
- Phenoxybenzamine may be used in a solution administered via NJ tube for better absorption and can successfully manage intraoperative blood pressures.
- Masses appearing to arise from the upper pole of the kidney or from the adrenal gland should be medically screened for pheochromocytoma.
- Surgical treatment for a giant pheochromocytoma may include both nephrectomy and adrenalectomy.

## Data Availability

Data sharing is not applicable to this article as no datasets were generated or analyzed during the current study.
